# Sodium Zirconium Cyclosilicate in the Gastrointestinal Tract Mimicking an Acute Gastrointestinal Bleed on CT

**DOI:** 10.3390/reports8020045

**Published:** 2025-04-10

**Authors:** John J. Hines, Joshua Roberts, Douglas S. Katz

**Affiliations:** 1 Department of Radiology, Huntington Hospital, Northwell Health, Huntington, NY 11743, USA; 2 Department of Radiology and Biomedical Imaging, Yale-New Haven Hospital, New Haven, CT 06510, USA; 3 Department of Radiology, NYU Winthrop Hospital, Mineola, NY 11501, USA

**Keywords:** CT scan, CT angiogram, GI bleed, lokelma, sodium zirconium cyclosilicate, hyperattenuating bowel contents

## Abstract

Hyperattenuating contents detected in the gastrointestinal (GI) tract on CT scans are commonly seen and are almost always due to the purposeful ingestion of an oral contrast agent, usually barium- or iodine-based, used for evaluating the GI tract. Occasionally, other ingested material such as antacids or other medications, foreign objects, and medical devices can also be hyperattenuating. While these are usually correctly identified, these materials can potentially be misdiagnosed as a pathologic condition. Lokelma (sodium zirconium cyclosilicate (SZC)) is an increasingly used agent to treat hyperkalemia and has a hyperattenuating appearance on CT due to the presence of zirconium. However, this is not well known to the radiologic community. Here, we describe a case where SZC was seen in the GI tract on CT and misinterpreted as an acute GI bleed. A 72-year-old woman underwent single (portal venous) phase intravenous contrast-enhanced abdominal and pelvic CT after presenting to the ED with a lower GI bleed. The CT showed intraluminal hyperattenuation within the cecum, which was diagnosed prospectively as an active GI bleed. A CT angiogram of the abdomen and pelvis performed the following day for follow-up showed the hyperattenuating contents to be present on the non-IV contrast-enhanced series of the study, thereby proving that it was not due to active bleeding. Further investigation of the patient’s medical record showed that the patient was being treated with SZC for hyperkalemia, accounting for the hyperattenuating cecal contents. Awareness of the hyperattenuating appearance of SZC on CT by radiologists and clinical staff can help avoid confusion and misdiagnosis.

Hyperattenuating contents in the gastrointestinal (GI) contents on CT examinations of the abdomen, pelvis, or chest are commonly encountered, and almost always due to the purposeful ingestion of an oral contrast agent, usually barium or iodine-based, employed to aid in the detection of gastrointestinal disease. Other less common sources of hyperattenuation in the lumen of the bowel include orally administered medications, such as antacids, foreign objects, and medical devices. Lokelma (sodium zirconium cyclosilicate (SZC)), a compound used to treat hyperkalemia, has a hyperattenuating appearance on CT and radiography due to the presence of zirconium, a transition metal in the periodic table. Here, we report a patient in whom SZC was seen in the GI tract as hyperattenuating material on CT, which was initially misdiagnosed as an acute GI bleed in the cecum. To our knowledge, this phenomenon and potential pitfall are not well known to radiologists.

A 72-year-old woman with multiple medical conditions, including end-stage renal disease on hemodialysis, presented to the Emergency Department with rectal bleeding. The patient was found to be hyperkalemic on admission (potassium of 5.7). The patient underwent a single-phase portal-venous phase CT scan of the abdomen and pelvis, performed with intravenous contrast and without positive oral contrast. The scan showed hyperattenuating contents in the cecum, which was interpreted by the attending radiologist to be contrast extravasation from an active gastrointestinal (GI) bleed (Figure 1a). There were no other acute or otherwise substantial findings on the scan, including no bowel wall thickening or mass. Based on this interpretation, a multi-phase CT angiogram of the abdomen and pelvis was performed the following day, in which similar intraluminal hyperattenuation, measuring 859 Hounsfield Units (HU), was seen in the cecum on the non-IV contrast enhanced series of the examination, performed prior to the administration of intravenous contrast, proving that this was not active bleeding (Figure 1b). The arterial and venous phase series from the CT angiogram showed no contrast extravasation anywhere in the GI tract to suggest an acute GI bleed (Figure 1c). Subsequent discussion with the clinical team caring for the patient revealed that the patient was being treated with SZC for her hyperkalemia, which explained the source of the hyperattenuating material in the bowel.

Positive oral contrast agents used for X-ray or computed tomography (CT) are usually barium- or iodine-based. Additionally, there is a wide spectrum of orally administered medications that are radiopaque on CT, including prescribed drugs, over-the-counter remedies, and multivitamins [1,2]. The hyperattenuating property of a drug can be due to a variety of substances, including but not limited to iodine, iron, bismuth, potassium, sulfur, aluminum, and calcium. Theoretically, any material with a high atomic number or mass density could be hyperattenuating. Rarely, hyperattenuating medications can be the source of misdiagnosis on radiography or CT, potentially being mistaken for gallstones, nephrolithiasis, gastric bleeding, and foreign objects [3,4,5,6,7]. Sodium zirconium cyclosilicate (SZC) is a relatively new medication, approved by the United States Food and Drug Administration in 2018. While it is hyperattenuating on various imaging modalities [8,9,10,11,12], there is a paucity of literature and a lack of knowledge among clinicians and radiologists.

Several recent case reports in the medical literature have shown SZC to be very dense on CT [8,9,10,11], with attenuation similar to routinely used barium- and iodine-based oral contrast agents and iodine-based intravenous contrast. Additionally, since SZC is administered as a liquid suspension, it will have a more diffuse distribution in the bowel than pill-based medications. It can thus mimic oral contrast, although the quantity of SZC in the bowel would be expected to be less than that of a routine oral contrast preparation used for abdominal and pelvic CT examinations. A review of the four prior case reports shows that SZC was visible as hyperattenuating liquid material throughout the proximal and distal gastrointestinal tract, including the stomach, small bowel, and colon. Kolesnik et al. CT-scanned a mixture of SZC with water (5 gms of SZC with 3 tablespoons of water), demonstrating high attenuation in vitro radiopacity of the suspension, with Hounsfield unit (HU) measurements ranging from 1040 to 1878 HU [8]. HU measurements of SZC in vivo may be lower, as in our case, due to dilution from ingested fluids or intestinal secretions, or potentially higher from intestinal fluid absorption.

Dual energy X-ray absorptiometry (DXA) results can also be affected by SZA. McCarney et al. showed significant alterations of bone mineral density measurements on DXA scans when scanning SZC solutions of varying concentrations with spine and body phantoms due to alterations in X-ray absorption by zirconium [12].

While the authors could not find any reports describing the appearance of SZC on conventional radiography, we identified SZC on a chest radiograph performed for the indication of shortness of breath in a patient on SZC treatment for hyperkalemia (Figure 2). Similar to the first patient described, there was no ingestion of hyperattenuating medications or oral contrast prior to the radiograph being performed, and therefore the hyperattenuating contents in the stomach were attributed to SZC.

SZC has been shown to effectively treat hyperkalemia with a low risk of hypokalemia and favorable tolerability and safety profiles compared to placebo. It is an inorganic, insoluble, non-polymer zirconium silicate compound that binds potassium and ammonium cations in the gastrointestinal tract in exchange for hydrogen and sodium cations, allowing for potassium excretion through the GI tract. The beneficial potassium-lowering effects can be utilized in patients with chronic kidney disease, heart failure, diabetes mellitus, and concomitant medications targeting the renin–angiotensin–aldosterone-system and maintained for up to 12 months [13]. It is not absorbed in the GI tract [14].

The hyperattenuating effect of SZC is due to zirconium, which has an atomic number of 40. Higher atomic number elements have more electrons with which photons may interact, as well as a denser electron cloud. They may therefore attenuate a higher proportion of the X-ray beam than lower atomic number elements. By comparison, the atomic numbers of barium and iodine are 56 and 53, respectively. Of note, the attenuation of SZC in the case patient was qualitatively similar to barium oral contrast and iodine-based oral and intravenous contrast agents on CT and did not cause any noticeable beam hardening artifact from its opacity.

Although the general radiological community may not be familiar with the imaging appearance of zirconium, it is commonly used in dentistry in the form of zirconia, an oxidized form of zirconium, which has metal and non-metal properties and is the basis for ceramic-based implants. The imaging appearance of zirconium and titanium-based implants and their potential to cause image-degrading artifacts are well documented in the dental literature [15,16,17].

The clinical utilization of SZC is on the rise, and radiologists are therefore more likely to encounter SZC in the GI tract on CT examinations. Knowledge regarding the imaging appearance of SZC is important, as it can be the cause of otherwise unexplained hyperattenuation in the GI tract on CT in a patient being treated for hyperkalemia, and a potential cause for confusion amongst radiologists and physicians from other specialties caring for the patient, as it did in our case and a previous report [10]. Moreover, SZC could potentially lead to misdiagnosis, as it initially did in our patient, in which the presence of SZC was mistaken for acute gastrointestinal hemorrhage. This is an important consideration in a patient where a CT examination is performed only with intravenous contrast-enhanced images. A CT angiography performed to evaluate acute GI bleeding typically consists of both non-enhanced and IV contrast-enhanced (usually arterial and more delayed phase) acquisitions. The non-enhanced series is essential to avoid misdiagnosis of commonly found hyperattenuating material, including surgical clips, sutures, stents and residual oral contrast from prior CT scans, as active bleeding, as it effectively did in our patient [18]. Alternatively, SZC may obscure the presence of intraluminal contrast extravasation in a patient experiencing acute GI bleeding, as has been previously reported in a case of duodenal hemorrhage related to peptic ulcer disease [11]. The high attenuation of SZC could also hinder the evaluation of mural abnormalities of the intestine, such as perforation, enteritis, ischemia or neoplasm, or could be mistaken for an ingested foreign object.

We present a case of hyperattenuating contents in the GI tract on a CT scan due to lokelma (sodium zirconium cyclosilicate), a relatively new potassium-lowering agent. Recognizing SZC as a potential source for hyperattenuation in the GI tract may help reduce confusion amongst clinicians and radiologists when seen on CT and could potentially avoid nondiagnostic or erroneous interpretations of CT scans for bowel-related disease.

## Figures and Tables

**Figure 1:**
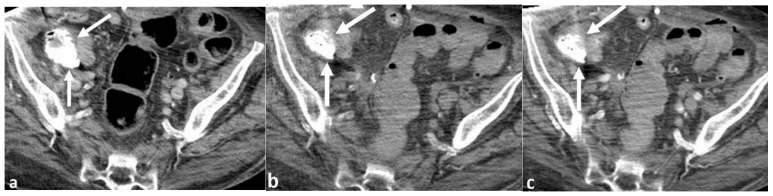
A 72-year-old woman with rectal bleeding. Axial intravenous contrast-enhanced image from single phase CT (**a**) shows hyperattenuating contents in the cecum (arrows), which was interpreted as active bleeding. Non-IV contrast (**b**) and arterial phase (**c**) images from the CT angiogram performed one day later again shows hyperattenuating contents (arrows), similar in volume and distribution to the previous CT scan and without change between the two series, proving that the hyperattenuating material was ingested material rather than intravenous contrast extravasation.

**Figure 2:**
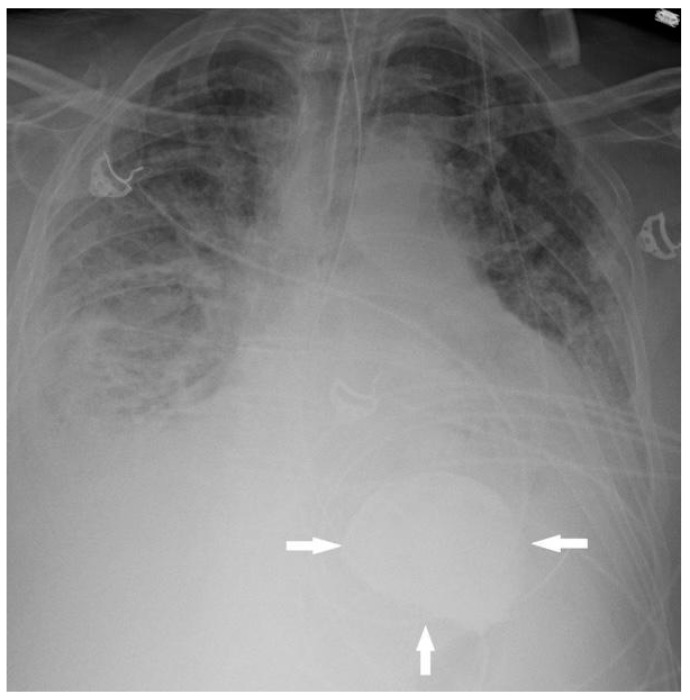
A 61-year-old man with shortness of breath. Chest radiograph showing hyperattenuating contents in the lumen of the stomach (arrows). After consultation with the intensive care physician caring for the patient, it was found that the patient was being treated for hyperkalemia with SZC, accounting for this finding.

## Data Availability

The original contributions presented in this study are included in the article. Further inquiries can be directed to the corresponding author.

## Abbreviations

The following abbreviations were used in this manuscript:

CT	Computed Tomography
SZC	Sodium Zirconium Cyclosilicate
GI	Gastrointestinal
DXA	Dual-energy X-ray absorptiometry
HU	Hounsfield Unit
